# Severe neonatal cytomegalovirus infection: about a case

**DOI:** 10.11604/pamj.2017.27.161.12004

**Published:** 2017-06-30

**Authors:** Brahim El Hasbaoui, Amal Bousselamti, Mohammed Amine Redouani, Amina Barkat

**Affiliations:** 1 Neonatal Medical and Resuscitation Department, Paediatrics V, Children’s Hospital, Faculty of Medicine and Pharmacy, University Mohammed V, Rabat, Morocco; 2 Research Team on Mother-child Health and Nutrition, Faculty of Medicine and Pharmacy, Mohamed V University of Rabat, Morocco

**Keywords:** Cytomegalovirus, congenital infection, petechiae, intrauterine growth retardation, ganciclovir

## Abstract

Maternofoetal infection with Cytomegalovirus (CMV) is the most common congenital infection and a leading cause of mental retardation and sensori-neural hearing loss. Population-based studies indicate that at least 0.5% of all infants born alive have CMV of whom approximately 10% have clinically evident symptomsat birth. The Justification of systematic screening for foetal CMV infection is still controversial and is not recommended in most developed countries. This is mainly justified by the paucity of antenatal prognostic factors and the lack of established intrauterine treatment when foetal infection has been diagnosed. In case of congenital CMV infection, infants can be symptomatic or asymptomatic at birth. Mortality for such infants can reach 30%, and survivors can have mental retardation, sensorineural hearing loss, chorioretinitis, and other significant medical problems. A newborn symptomatic is defined by the existence of clinical and / or biological signs and / or neonatal imaging, the most frequent clinical signs are: hepatosplenomegaly (60%), microcephaly (53%), jaundice (67%), petechiae (76%), at least one neurological abnormality (68%). The frequency of biological abnormalities is as follows: increase in transaminases (83%), thrombocytopenia (77%), hyperbilirubinemia (69%), haemolysis (51%), hyperproteinorrachy (46%). The abnormalities of neonatal imaging are present in 70% of symptomatic newborns; intracerebral calcifications are the most frequent abnormalities. We report a case of newborn who presented a congenital infection by CMV, evoked on the intrauterine growth retardation, organs of the reticulo endothelial and haematological system were reached while nervous system was spared, and CMV PCR was very positive. indicating an antiviral treatment for 6weeks based on ganciclovir.

## Introduction

Maternal-foetal CMV infection is the leading infectious cause of congenital malformation, mental retardation and deafness [1] and affects about 1% of births [2]. Preventive (hygienic) measures [3] represent the best weapon to combat the occurrence of this infection. Immunization prevention (not currently marketed) [4] and the use of intravenous specific immunoglobulins (not yet available in most countries) [5] may become, in the near future, other important factors influencing the incidence of this disease. Systematic screening is not proposed except rarely by individual doctors. The CMV being sought either on a sign of ultrasound call or in case of maternal fever. Indeed, in France, National Agency for Health Accreditation and Evaluation (Anaes) doesn't recommend the systematic screening of the CMV because of: The lack of a reliable marker for prognosis in prenatal CMV infection; The absence of reliable preventive or curative treatment of infection and materno-foetal transmission; And the fact that infected foetuses are, for the most part, “asymptomatic” at birth. There are few reports of the treatment intervention with the antiviral medication for the neonatal cytomegalovirus infection of mature neonates. In case of congenital CMV infection, it has been reported that prompt treatment intervention with antiviral agents for 6 weeks reduces the severity of complications [6]. However, there is no consensus guide to medical care and differentiation from congenital CMV infection for CMV infection in the early stage of a newborn period. We report a case of newborn who presented a congenital infection by CMV, evoked on the intrauterine growth retardation, organs of the reticulo endothelial and haematological system were reached while nervous system was spared, and CMV PCR was very positive indicating an antiviral treatment for 6weeks based on ganciclovir.

## Patient and observation

Patient was born as preterm 36WA male baby with body weight of 1700g, during the perinatal period the Newborn presented an intrauterine growth retardation and alteration of cerebral vascularization, indicating a Caesarean Section (C-Section) Birth and Delivery with good adaptation to extra-uterine life. The newborn was admitted in our department at day one of life for prematurity of 36 SA, with intrauterine growth retardation, diffuse jaundice and petechiae were found on face, trunk and limbs with hepatosplenomegaly. Laboratory data showed; anaemia Hb 12,2g/dl, leukopenia 8510/μL, platelet was markedly decreased to 18,000/μL, hepatic cytolysis; ALAT 159U/L; ASAT 140U/L; total bilirubin 151mg/l; Direct bilirubin 109mg/l; and biological cholestasis; alkaline phosphatase (ALP) of 174; Gamma glutamyltransferase of 252UI/L activated partial thromboplastin time (PTT) was normal;Prothrombin Time (PT) of 50%. Abdominal ultrasound has noticed hepatosplenomegaly while the Brain CT revealed no calcification or other abnormal findings such as ventricular enlargement. Because of CMV PCR was very positive (54305UI/ml) in the peripheral blood, intravenous ganciclovir administration (15 mg/kg/day) was started and blood transfusion was given for his anaemia. After 4 weeks of treatment, the evolution was marked by a good clinical and biological improvement with regression of jaundice (Direct bilirubin at 47mg/ml; total bilirubin at 63mg/l) and petechiae, betterment of liver function (ALAT 56UI/L; ASAT 83UI/L, platelet count was increased rapidly to normal level (platelet at 165000/μL) while Viral load of CMV was decreased to 572UI/ml. The newbornhas subsequently presented a reappearance of jaundice and petechiae, laboratory data showed: Hb 10g/dl, leukopenia 2750/μL, platelet at 73000/μL, ALAT 91U/L; ASAT 123U/L; total bilirubin 101mg/l; Direct bilirubin 77mg/l, with a rescinding of the viral load (54305UI/ml)making suspected a resistanceto ganciclovir or a reactivation of CMV in relation with breastfeedingtherefore we had switched to an oral antiviral drug (valganciclovir 30 mg/kg/day) for fourth week. Unfortunately the newborn died as a result of a lightning hemorrhage(Hematemesis, epistaxis, otorrhagia).

## Discussion

With an estimated prevalence of 0.6% in newborn infants, human cytomegalovirus (CMV) is the most common congenital infection worldwide and a leading cause of childhood hearing loss, cognitive deficits, [7] and vision impairment. In spite of the detailed knowledge about the epidemiology and pathogenesis of CMV infections maternal, fetal and neonatal, the screening for CMV infection in pregnancy remains controversial. Vertical transmission of CMV to the fetus can occur during pregnancy, at delivery, or after birth through exposure to breast milk, because of its nutritional and non-nutritional benefits, human milk is recommended for both preterm and term-born infants [8]. At the same time, human milk is a prominent mode of CMV transmission in early postnatal life of preterm infants [9]. The incidence of CMV reactivation in mothers during lactation is high, particularly following premature birth [10]. In CMV infections among pregnant women, the gold standard of serologic diagnosis is maternal seroconversion based on the detection of IgG antibodies to CMV. The IgG assay is nearly 100% sensitive and specific, readily available, and automated for high volume capacities [11]. The diagnosis via seroconversion is seldom achieved since an initial seronegative serum is rarely available. We and others have observed that IgM usually peaks 3 to 6 months after a primary infection but may remain present in serum for over 12months [12]. Hence, finding IgM to CMV in a single serum of a pregnant woman does not alone establish a recent primary CMV infection during pregnancy [13]. Antibody avidity, which is an indirect measure of the tightness of antibody binding to its target antigen, increases in the first weeks after a primary infection. Low avidity IgG antibodies to CMV persist for up to 20 weeks after a primary CMV infection [14]. These low avidity antibodies are then replaced by high avidity antibodies (>60% binding in presence of 5M urea). Currently, the combination of the presence of anti-CMV IgM antibodies and low avidity anti-CMV IgG antibodies along with maternal or fetal symptoms is used for the diagnosis of a primary maternal infection [15].

The diagnosis of fetal infection via Amniotic fluid is a helpful adjunct in maternal diagnosis but cannot replace maternal serologic testing because amniotic fluid may contain CMV even if the mother was immune to CMV before conception. The best test for the diagnosis of intrauterine infection is detection of CMV in the amniotic fluid by culture and PCR. One of the first studies observed that amniocentesis correctly identified 12 of 13 (92%) infants with congenital CMV infection [16]. In our case, the screening for CMV infection has not been performed. In case of congenital CMV infection, Infants can be symptomatic or asymptomatic at birth. Symptomatic infections are seen in approximately 10% of infants with congenital CMV infection; these infants suffer substantially. Mortality for such infants can reach 30%, and survivors can have mental retardation, sensorineural hearing loss, chorioretinitis, and other significant medical problems [17]. Congenitally infected newborns who are asymptomatic at birth also can have significant sequelae, with as many as 15% developing sensorineural hearing loss.3 In contrast, infants infected with CMV perinatally and postnatally generally do well. The definition of an infected newborn is based on the detection of the virus or its genome in the urine. A viral search can also be performed on the saliva or blotting paper used for the Guthrie test [18]. Infected newborns may be symptomatic or asymptomatic. A newborn symptomatic is defined by the existence of clinical and / or biological signs and / or neonatal imaging. These signs are summarized in Table 1 [19]. The most frequent clinical signs are: Hepatosplenomegaly (60%), microcephaly (53%), Jaundice (67%), petechiae (76%), at least one neurological abnormality (68%) [20]. In our case, the newborn presented prematurity of 36 SA, with intrauterine growth retardation, diffuse jaundice and petechiae were found on face, trunk and limbs with hepatosplenomegaly. The frequency of biological abnormalities is as follows: increase in transaminases (83%), thrombocytopenia (77%), hyperbilirubinemia (69%), haemolysis (51%), hyper-proteinorrachy (46%) [20]. In our case, we had: anaemia, leukopenia, platelet was decreased to 18,000/μL, hepatic cytolysis and biological cholestasis. The abnormalities of neonatal imaging (transfontanellar ultrasound, cerebral scan) are present in 70% of symptomatic newborns. Intracerebral calcifications are the most frequent abnormalities.In our case there was no neurological involvement.

**Table 1:** Clinical and biological abnormalities in neonatal cytomegalovirus infection

Clinical signs	Biological Signs
Prematurity	Increased transaminases> 80 U / L
Hypotrophy	Thrombocytopenia <100,000 / mm^3^
Petechies, purpura	Hyperbilirubin conjugated
Jaundice	Hemolysis
Hepatosplenomegalia	Increase in proteanorachia> 120 mg / gL
Microcephaly	
Hypotonia	
Suction disorder	
Convulsions	
Ophthalmic anomaly	
Pneumopathy	
Dental anomaly	

Approximately 10% of infected newborns are symptomatic. The infection involves several organs with a predilection for the reticuloendothelial system and the central nervous system (CNS). Approximately half of these newborns present the classic forme of cytomegalic inclusions disease. The other half has moderate or atypical signs. It is estimated that the mortality rate in this group “Symptomatic” is 5-10% [21]. From survivors, the sequelae rate is 90%. In 70% of cases, psychomotor retardation is accompanied by anomalies neurological disorders and microcephaly. A hearing loss occurs in 50% of cases; Most often bilateral and, in half of the cases, progressive. Optic atrophy or chorioretinitis is objective in 20% of cases. The predictive factors of adverse neurological prognosis are: microcephaly, chorioretinitis, the presence of any other clinical neurological abnormalities at birth, and the presence of cerebral abnormalities detectable by transfanellar or cerebral scan in the first month of life [22]. Boppana et al. showed that, in 56 infected children, almost 90% of children with brain-scan abnormalities had at least one type of sequelae compared to 29% for those with no imaging abnormalities [23]. Rivera et al. Showed by multivariate analysis that the presence of growth retardation at birth as well as petechiae were independent prognostic factors of the occurrence of hearing deficiency [24]. Though, about 90% of infected newborns are asymptomatic. The prognosis of these children is clearly better than for symptomatic newborns. However, it is estimated that about 10-15% of these children will have sequelae. In a recent study of infected neonates, it has been shown that the mean values of viral neonatal blood load are statistically higher in neonates who developed sequelae than in those who did not and that almost 70% of sequelae in newborns have were observed when the viral load was greater than 10,000 copies per 100,000 leukocytes [25]. The viral load in fetal blood could therefore be an important prognostic factor, and further studies are needed to assess its predictive value. Treatment of CMV infections has developed significantly over the past 20 years, and four molecules are currently available: Valaciclovir which is used in the prophylaxis of CMV infection and ganciclovir, foscarnet and cidofovir which are used in cure. These molecules, which are targeted to phosphotransferaseviral (ganciclovir) and / or viral DNA (ganciclovir, foscarnet and cidofovir) are virostatic and do not allow the organism to eradicate CMV. In addition, these are highly toxic molecules. Because infants with symptomatic congenital CMV infection have the greatest mortality and long-term morbidity, evaluation of antiviral therapy initially has focused on such infants. Studies conducted by the National Institute of Allergy and Infectious Diseases Collaborative Antiviral Study Group (CASG), showed that infants with congenital CMV infection with central nervous system involvement. Receiving 6 weeks of parenteral ganciclovir therapy. The majority of these infants, as in our case, had thrombocytopenia and neutropenia attributed to the ganciclovir therapy. Although the amount of CMV excreted in urine decreased during therapy, viruria returned to near pretreatment levels after therapy was stopped. Hearing improvement or stabilization was evident in 16% of 30 infants at 6 months of age.

## Conclusion

Congenital cytomegalovirus infection is frequent. Symptomatic disease at birth is infrequent but very severewithhigh mortality rate. In most cases the infection is totally asymptomatic but neurosensorial damage can occur in 10% to 15% of children, hearing loss being the most frequent. Maternal or neonatal screening is the only way to recognise asymptomatic disease. Our work illustrates that even if maternal fetal infection with CMV remains asymptomatic in most of cases it may have serious or even fatal fetal or neonatal complications, resulting in early screening and treatment in women pregnancy, underlining the importance of vaccine prevention and the use of intravenous specific immunoglobulins could become, in the near future, important factors influencing the incidence of this disease.

## Competing interests

The authors declare no competing interest.
